# Integrated Metabolome and Transcriptome Analysis Uncovers the Role of Anthocyanin Metabolism in *Michelia maudiae*

**DOI:** 10.1155/2019/4393905

**Published:** 2019-11-03

**Authors:** Xiaoan Lang, Na Li, Lingfei Li, Shouzhou Zhang

**Affiliations:** Key Laboratory of Southern Subtropical Plant Diversity, Fairy Lake Botanical Garden, Shenzhen & Chinese Academy of Sciences, Shenzhen 518004, China

## Abstract

*Michelia maudiae* Dunn is one of the important ornamental plants in the Magnoliaceae family, and the color of its flowers usually appears naturally pure white. The discovery of a rubellis flower named *M. maudiae* Dunn var. *rubicunda* provides an opportunity to reveal the metabolism of the flavonoids and anthocyanins of this “early angiosperm” plant. Combined metabolome and transcriptome analyses were applied using white and rubellis mutant tepals. Seven stages have been divided for flower development, and forty-eight differentially altered metabolites were identified between white and rubellis tepals at a later stage. The major anthocyanins including peonidin *O*-hexoside, cyanidin *O*-syringic acid, cyanidin 3,5-*O*-diglucoside, cyanidin 3-*O*-glucoside, and pelargonidin 3-*O*-glucoside were upregulated over 157-fold in the mutant. Conversely, the highly significant accumulation of the colorless procyanidin or the slightly yellow epicatechin and catechin was found in white flowers. Putative homologues of color-related genes involved in the phenylpropanoid and flavonoid biosynthesis pathway were identified in the transcriptome. The increasing expression of *dihydroflavonol 4-reductase* (*DFR*) might play an important role in the occurrence of rubellis pigments, while the overexpression of *anthocyanidin reductase* (*ANR*) in white flowers may promote the biosynthesis of proanthocyanidins. Additionally, several coloration-related repressor R2R3-MYB transcription factors showed different expression levels in the tepals of the rubellis mutant. This study provides a comprehensive analysis relating color compounds to gene expression profiles of the Magnoliids plant *M. maudiae*. The newly generated information will provide a profound effect on horticultural applications of Magnoliaceae.

## 1. Introduction

Flower coloration is one of the most attractive characteristics and quality traits of ornamental plants and has the ability to attract pollinators and seed distributors. Moreover, a broad spectrum of colors also provides an important aesthetic function for plants, especially in horticulture applications. Flower color is largely determined by the production of pigments, usually anthocyanins, betalains, or carotenoids. Anthocyanins are major contributors to flower colors. The primary shade of a flower color is mainly determined by the ratio of three different classes of anthocyanidins, namely, pelargonidin (orange to brick red), delphinidin (purple to blue), and cyanidin (red to pink to blue), and subsequent modifications to structure such as glycosylation, methylation, and acylation [1]. Anthocyanin biosynthesis has been extensively studied in various horticultural plants, for example, *Chrysanthemum grandiflorum* [2], *Begonia semperflorens* [3], and *Matthiola incana* [4]. Two major classes of genes are required for anthocyanin biosynthesis: structural genes and transcription factors (TFs). The structural genes encode the enzymes that are responsible for the biochemical reactions of anthocyanin synthesis. Anthocyanin biosynthesis starts from phenylalanine and produces colorless secondary intermediate metabolites that are sequentially catalyzed by a number of enzymes, including phenylalanine ammonia-lyase (PAL), cinnamic acid 4-hydroxylase (C4H), 4-coumarate-CoA ligase (4CL), chalcone synthase (CHS), chalcone isomerase (CHI), flavanone 3-hydroxylase (F3H), flavanone 3′-hydroxylase (F3′H), flavanone 3′5 ′-hydroxylase (F3′5′H), and dihydroflavonol 4-reductase (DFR) [5]. The colorless leucoanthocyanidins are then transformed into stable-colored anthocyanins by anthocyanidin synthesis (ANS) and UDP-glucose flavonoid 3-*O*-glucosyltransferase (UFGT) resulting in a series of flower color polymorphisms [6]. The TFs control the expression of the structural genes during anthocyanin biosynthesis. There are evidences that TFs MYB, basic helix-loop-helix (bHLH), and WDR play an important role in regulating the activity of the structural genes [7–9]. The coordinated expression of the structural genes and TFs lead to the accumulation of anthocyanins resulting in color variation.

The color mutants provide important materials to unravel the complex mechanism of flower pigmentation. The detailed knowledge of the events of the biosynthesis pathway is based on the research with color mutation forms of *Zea mays*, *Petunia hybrida*, and *Arabidopsis thaliana* [10–12]. In *Arabidopsis*, MYB75, MYB90, and bHLH have been reported to regulate the expression of *DFR* and *ANS* genes, thus affecting the biosynthesis of anthocyanin [10]. In *Vaccinium myrtillus*, *PAL*, *CHS*, *F3H*, *DFR*, and *ANS* are all involved in color mutant forms [5]. In *Muscari armeniacum*, flavonol synthase (FLS) and DFR are responsible for blue pigmentation [13]. In recent years, combined large-scale sequencing analyses have been used to lucubrate on the pigmentation of flowers or fruits [3, 14, 15]. Integrated metabolome and transcriptome analyses have elucidated the expression of differentially expressed genes (DEGs) and the respective metabolic products [13, 16]. It is possible to gain a comprehensive analysis relating metabolites to gene expression profiles and get an insight into the variation mechanisms, which would not be possible from transcript analysis or metabolomic analysis alone.

**Figure 1:**
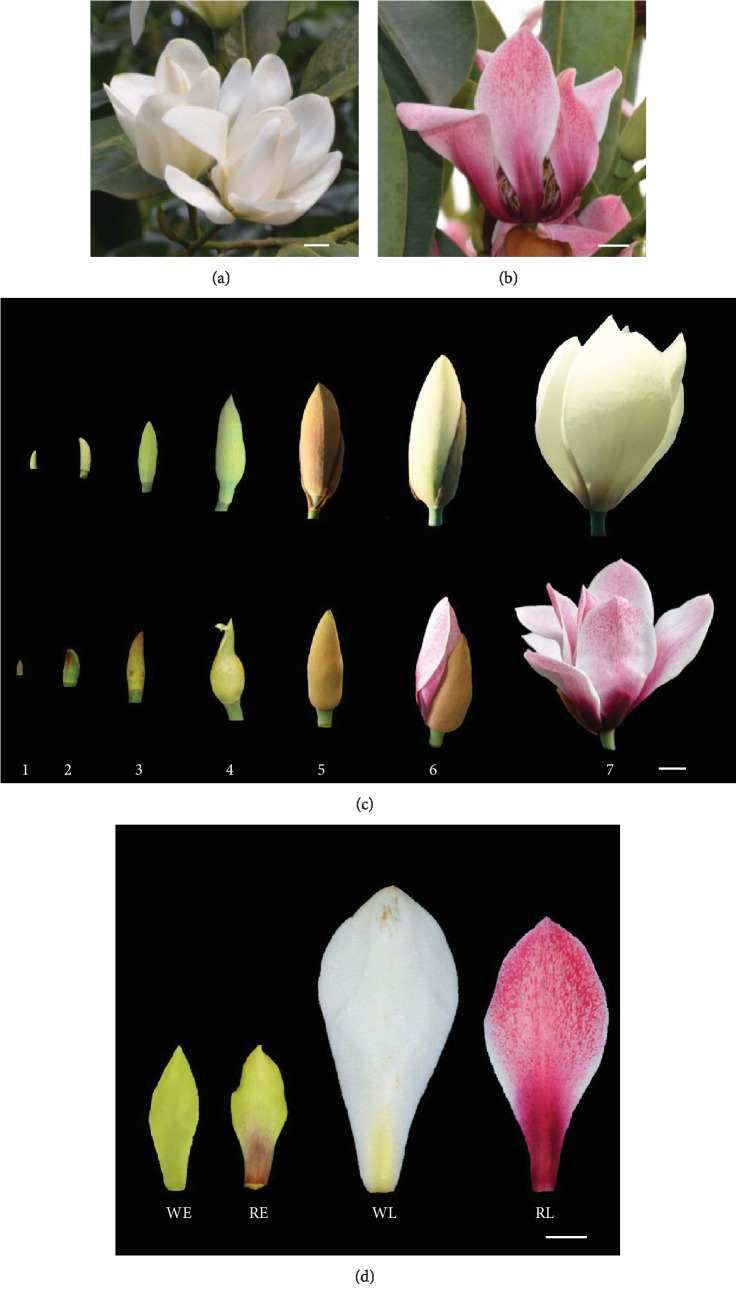
Flower morphology of *Michelia maudiae*. (a) “White” flower of *M. maudiae* Dunn. (b) “Rubellis” flower mutation of *M. maudiae* Dunn var. *rubicund*. (c) Different developmental stages of the two types of *M. maudiae*. (d) Definition and characterization of experimental materials for transcriptome and metabolome data. WE: “white flower” at an early stage (stage 3); WL: “white flower” at a later stage (stage 6); RE: “Rubellis flower” at an early stage (stage 3); RL: “Rubellis flower” at a later stage (stage 6).

Magnoliaceae, a primitive family of flowering plants, plays an important role in the studies of biogeography and evolution [17]. Moreover, these plants are widely appreciated as ornamental shrubs and trees because the trees have a graceful appearance and produce beautiful flowers and they attract a great deal of attention from horticulturalists. *Michelia maudiae* Dunn., one species of Magnoliaceae, is a broad-leaved evergreen tree that has a fleshy stem that profusely branches and bears white flowers; it is popular as an ornamental plant in East and Southeast Asia, Central America, Southeast North America, and South America [18]. The natural flower color of *M. maudiae* is pure white (Figure 1(a)). In 2009, a *M. maudiae* var. *rubicund* with a rubellis flower was found by Fan et al. in the wild [19] (Figure 1(b)). The variation of flower color in *M. maudiae* provides a higher ornamental value in the family of Magnoliaceae. However, the mechanism of flower pigmentation in the family Magnoliaceae is still far from conclusive.

In this study, metabolome and transcriptome analyses were performed using the early and later stages of the white and rubellis flowers of *M. maudiae*. Beyond identifying many differentially altered metabolites, the corresponding transcriptional changes of structural genes and TFs in the phenylpropanoid and flavonoid biosynthesis pathways have also been revealed. Our research provides valuable information to further elucidate the molecular basis of flower color formation in *M. maudiae* and to further elucidate the novel gene resources for Magnolia plant breeding.

## 2. Materials and Methods

### 2.1. Plant Materials

*M. maudiae* flowers were divided into seven stages according to the length of the tepals and the main characters of the flowers (Figure 1(c), [Supplementary-material supplementary-material-1]). The tepals of *M. maudiae* from the early stage (stage 3) were collected at 10:00 a.m., November 2015, and the tepals from the later stage (stage 6) were collected at 10:00 a.m., in March 2016. Tepals from both stages were taken from the Xi'an Botanical Garden, Shaanxi Province, China. Three biological replicates were collected per sample, each with 30 representative tepals randomly selected from several trees in the garden. All samples were immediately frozen in liquid nitrogen and stored at -80°C for further studies.

### 2.2. Identification and Quantification of Anthocyanin Compositions

The anthocyanin compositions of the two stages of rubellis and white tepals were determined using high-performance liquid chromatography (HPLC) with three biological replicates. The freeze-dried tepals were crushed and 100 mg powder was extracted overnight at 4°C in 1.0 ml 70% methanol. After centrifugation at 10,000*g* for 10 min, the supernatant was filtrated through 0.22 *μ*m pores and awaited HPLC and mass spectrometry (LC-MS) analysis. A quality-control sample was made by equal blending of all samples and was run after every 10 injections to ensure the stability of the analytical conditions.

The filtered supernatant was injected into a HPLC system (Shim-pack UFLC SHIMADZU CBM30A, Kyoto, Japan) equipped with a Waters ACQUITY UPLC HSS T3 C18 column (1.8 *μ*m, 2.1 mm × 100 mm; Waters, Milford, MA, USA). The solvent system was ultrapure water (0.04% acetic acid) and acetonitrile (0.04% acetic acid). The gradient program was 100 : 0 at 0 min, 5 : 95 at 11.0 min, 5 : 95 at 12.0 min, 95 : 5 at 12.1 min, and 95 : 5 at 15.0 min. The flow rate was set at 0.40 ml/min. The temperature of the column was maintained at 40°C. The effluent was then scanned using an ESI-triple quadrupole-linear ion trap-MS/MS system (Applied Biosystems 4500 Q TRAP) as described previously [16, 20]. The results were further analyzed by annotating against public databases, including MassBank (http://www.massbank.jp/), HMDB (http://www.hmdb.ca/), KNAPSAcK (http://www.knapsackfamily.com/KNApSAcK/), METLIN (http://metlin.scripps.edu/index.php), and MoToDB (http://www.ab.wur.nl/moto/) to identify metabolites. Metabolite quantification was carried out using MRM (multiple reaction monitoring) [21]. Partial least squares-discriminant analysis (PLS-DA) was performed with the identified metabolites [22]. Compositions with fold change ≥ 2 or ≤0.5 and the thresholds of variable importance in project (VIP) ≥ 1 were defined as significant-difference metabolites.

### 2.3. RNA Extraction, Library Construction, and Transcriptome Sequencing

Total RNAs from the stage 3 and stage 6 of rubellis and white tepals were extracted using a plant RNA kit following the manufacturer's protocol (Omega Bio-tek Inc., Doraville, GA, USA). To confirm the reliability of the RNA-sequencing experiments, three replicates of the samples per developmental stage of the same color tepals were equally blended to obtain RNA extracts. The quality of RNA was evaluated using Bioanalyzer 2100 (Agilent Technologies Inc., CA, USA). Those samples with an RNA integrity number (RIN) above 6.6 were used for the preparation of cDNA libraries.

A total amount of 1 *μ*g RNA per sample was used to input material for library construction. Sequencing libraries were generated using Illumina's NEBNext®Ultra™ RNA Library Prep Kit (NEB, USA) according to the manufacturer's instructions. Briefly, mRNA was purified from total RNA using oligo (dT) magnetic beads. And then, mRNA was fragmented into small pieces by the addition of NEBNext First-Strand Synthesis Reaction Buffers (5×) under high temperature. Secondly, the fragments served as templates to synthesize first-strand cDNA using random hexamer primers and M-MuLV reverse transcriptase. Second-strand cDNA was subsequently synthesized using DNA polymerase I and RNaseH. Lastly, to preferentially select cDNA fragments 150~200 bp in length, the library fragments were purified with the AMPure XP system (Beckman Coulter, Beverly, USA). The size-selected, adaptor-ligated cDNA was used as templates for PCR amplification and assessed using the Bioanalyzer 2100 system. Four libraries (early and later tepals of rubellis flowers and early and later tepals of white flowers) were sequenced on the Illumina HiSeq™ 2000 platform. The stage 3 and stage 6 in *M. maudiae* Dunn are termed as “white flower” at early stage (WE) and later stage (WL), respectively. The stage 3 and stage 6 in the mutant are noted as “rubellis flower” at early stage (RE) and later stage (RL), respectively.

### 2.4. Transcriptome Assembly and Functional Annotation

Clean reads were obtained by removing adaptor sequences and low-quality reads from raw data and using these for all the subsequent analyses. Q20 and Q30 values and GC content of the clean data were calculated. Clean reads from all libraries were pooled together and then assembled using the software Trinity v2.8.0 [23, 24] with min_kmer_cov set to 3. To annotate the *M. maudiae* transcriptome, assembled sequences were aligned against the public dataset of nonredundant (Nr) databases, nucleotide sequence (Nt) database, the Swiss-Prot protein sequence database (SWISS-PROT), the Kyoto Encyclopedia of Genes and Genomes (KEGG) database, Gene Ontology (GO), and the Clusters of Orthologous Groups of proteins (COG) databases, respectively, using BLASTX with a threshold *E* value of ≤1 × 10^−5^ [25–27].

### 2.5. Differential Expression Analysis of Unigenes

Clean reads of each sample were mapped back to the assembled transcriptome by expectation maximization [28]. And then for FPKM, the fragments per kb per million reads of each unigene was calculated for estimating gene expression levels based on the mapping results [29]. Differential expression analysis of two samples was performed using the DEGseq R package [30]. DEGseq estimates differential expression from sequencing data based on the negative binomial distribution. The resulting *Q* values were adjusted using the Benjamini-Hochberg method for controlling the false discovery rate (FDR) [31]. Genes with the criterion of *Q* value < 0.01 (cut-off at 5% FDR) screened by DEGseq and an absolute value of log_2_ ratio ≥ 2 were assigned as differentially expressed. GO and KEGG functional enrichment analysis of the DEGs was implemented using the GOseq R package based on the Wallenius noncentral hypergeometric distribution [32].

### 2.6. Phylogenetic Analysis

Putative R2R3-MYB sequences were extracted and aligned with the closely related R2R3-MYB protein using the program ClustalW. MEGA version 7 (MEGA7) was applied for phylogenetic inference. Maximum likelihood phylogenies were estimated using amino acid sequence alignments. The following GenBank accession numbers were used: AAS68190 (VvMYB5a), Q58QD0 (VvMYB5b), AAG42001 (AtMYB75), AM259485 (VvMYBA1), ACK56131 (VvMYBPA2), Q9FJA2 (AtMYB123), AAK84064 (FaMYB1), ACX50288 (VvMYBC2-L2), AHX24372 (PhMYB27), ABW34393 (VvMYBC2-L1), P81395 (AmMYB330), NP_849749 (AtMYB8), NP_192684 (AtMYB6), NP_179263 (AtMYB7), NP_195225 (AtMYB32), AAC83582 (AtMYB4), NP_001106009 (ZmMYB42), ADX33331 (PhMYB4), CAE09058 (EgMYB1), AEM17348 (PvMYB4a), ABL61515 (VvMYB4a), ACN94269 (VvMYB4b), and NP_001105949 (ZmMYB31).

### 2.7. Gene Validation and Expression Analysis

To validate the transcriptional abundance results from sequencing analysis, nine color-related unigenes were subjected to real-time quantitative PCR analysis. A total of 1 *μ*g RNA was reverse transcribed to first-strand cDNA using the HiScript® III RT SuperMix for qPCR kit (Vazyme Biotech Co., Ltd., China) according to the manufacturer's instructions. The reaction product was diluted 20-fold with sterile water for RT-qPCR analysis using a ChamQ™ Universal SYBR® qPCR Master Mix (Vazyme Biotech Co., Ltd., China). Real-time PCR was performed on a CFx96™ instrument (Bio-Rad, USA). The actin gene was used as the internal control for the normalization of gene expression. Primers were designed using Primer Premier 5 and listed in [Supplementary-material supplementary-material-1]. The RT-qPCR reactions were carried out in 20 *μ*l volumes containing 0.4 *μ*l of each primer (concentration of 10 *μ*M), 10 *μ*l of ChamQ Universal SYBR qPCR Master Mix, and 2 *μ*l cDNA. The RT-qPCR cycling conditions were as follows: 95°C for 30 s, followed by 40 cycles of 95°C for 10 s, and 60°C for 30 s. A melting-curve analysis was performed with the default setting of the instrument. Relative expression was calculated by the 2^−*∆∆*CT^ method. Values for mean expression and standard deviation were calculated from the results of three biological replicates.

## 3. Results

### 3.1. Developmental Stage Division and Morphological Description of *M. maudiae* Flowers

For the convenience of further study, we divided *M. maudiae* flowers into seven developmental stages according to the length of the tepals and the main characteristics of the flowers ([Supplementary-material supplementary-material-1], Figure 1(c)). The pigmentation of the tepals begins at stage 3 and reaches the maximum level at stage 6, just ready to open (Figure 1(c)). At stage 3, both the tepals of the white flower and its rubellis mutant have a green appearance, with a slight rubellis at the bottom of the tepals in the rubellis mutant (Figure 1(d)). At stage 6, the flowers are about to bloom for a very short time, with superne rubellis and inferne atrophoeniceis in the tepals of the rubellis mutant (Figure 1(d)).

### 3.2. Metabolome Composition Analyses in *M. maudiae*

To examine the biochemical mechanism of the appearance of the rubellis color phenotype in *M. maudiae*, the metabolomic profiles of the tepals were compared. Firstly, the total contents of anthocyanins in the tepals of two strains were detected by a spectrophotometer ([Supplementary-material supplementary-material-1]). As expected, total anthocyanins were highly accumulated in RE and RL but not in WE, and only a bit was detected in WL. The result corresponded well with the pigmentation of flowers in *M. maudiae*.

Furthermore, to gain additional information on the coloration of *M. maudiae* flowers, the general secondary metabolites were identified in the samples WL and RL. The metabolite profiles of WL and RL showed significant differences ([Supplementary-material supplementary-material-1]). A total of 149 metabolites were identified from these two samples with three biological replicates, among which 48 differentially altered metabolites (DAM) were identified, including 7 anthocyanins, 5 catechin derivatives, 2 flavanone, 17 flavones, 4 flavonols, 3 isoflavones, 8 flavone *C*-glycosides, and 2 proanthocyanidins (Table 1).

Almost differentially altered anthocyanins demonstrated significantly higher contents in the later stage of rubellis tepals. Compared with white tepals, peonidin *O*-hexoside was found with 3,381-fold increments; cyanidin 3-*O*-glucoside and cyanidin 3,5-*O*-diglucoside were detected with 157-fold and 322-fold increments, respectively; and cyanidin *O*-syringic acid and pelargonidin 3-*O*-glucoside were detected at significantly higher concentrations in rubellis flowers but at barely detectable levels in white flowers, which could explain the rubellis color of the mutant. Surprisingly, one derivative of cyanidin, rosinidin *O*-hexoside, was detected at significantly higher levels in white tepals but was detected at a level so low as to barely be detectable in rubellis tepals (Table 1).

The derivatives of catechin are also widely distributed in plants. Catechin and epicatechin were the two major catechin derivatives presented in white tepals, while the rubellis-flowered strain contained epigallocatechin, protocatechuic acid, and protocatechuic aldehyde with significantly higher levels (Table 1).

Compared to the color components in rubellis flowers, the colorless A- and B-type procyanidins were markedly upregulated in white flowers (Table 1). Procyanidin B3 in WL was upregulated over 25-fold compared to that in RL. The content of procyanidin A2 was far greater in white flowers, but the content was barely detectable in rubellis tepals.

In addition, the rubellis tepals also contain several flavones, flavonols, and isoflavones. These components are either colorless or slightly yellow. For the flavones, several derivatives of tricin, including tricin *O*-vanilloyl hexoside, tricin di-*O*-hexoside, tricin *O*-rhamnoside, tricin *O*-hexosyl-*O*-syringin alcohol, and tricin 5-*O*-hexoside, were remarkably upregulated in the rubellis tepals vs. the white tepals. In addition, an intermediate in the tricin biosynthesis, tricetin *O*-rutinoside, was also markedly upregulated in the rubellis tepals. Hence, tricin may play an essential role in rubellis tepals.

### 3.3. De Novo Assembly and Functional Annotation

To further elucidate the matter on *M. maudiae* discussed above at the molecular level, four libraries from stage 3 and stage 6 of white and rubellis tepals (WE, WL, RE, and RL) were conducted, resulting in approximately ~41 million clean reads for each sample ([Supplementary-material supplementary-material-1]). The Q20 and Q30 of each sample were greater than or equal to 96.9% and 93%, respectively. The GC contents accounted for ~47% of these reads. De novo assembly of these clean reads resulted in 109,729 nonredundant unigenes with a mean length of 925 bp ([Supplementary-material supplementary-material-1]). The N50 value was 1,611 bp and 90% had a length of at least 352 bp. All these data indicated that the sequencing quality was high enough for further study.

Nearly 37.64% of unigenes had top matches to sequences from *Nelumbo nucifera*. The other five top-hit species were *Vitis vinifera*, *Elaeis guineensis*, *Phoenix dactylifera*, *Amborella trichopoda*, and *Gossypium raimondii* ([Supplementary-material supplementary-material-1]). Based on GO annotation, 96,164, 58,324, and 35,013 unigenes were successfully assigned into three main GO categories: biological process, cellular component, and molecular function ([Supplementary-material supplementary-material-1]). A search against the COG database resulted in the classification of 39,811 unigenes into 25 COG categories ([Supplementary-material supplementary-material-1]). The maps with the highest unigene representation were “General function prediction only” (6,540 unigenes), followed by “Transcription” (3,898 unigenes), “Replication, recombination, and repair” (3,566 unigenes), “Signal transduction mechanisms” (2,811 unigenes), and “Posttranslational modification, protein turnover, and chaperones” (2,837 unigenes).

**Figure 2:**
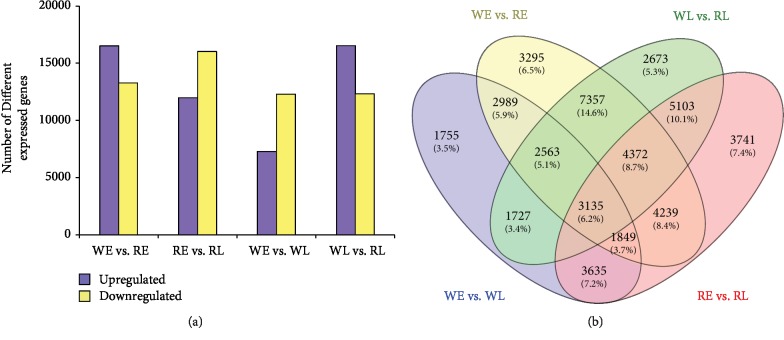
Differentially expressed genes between early and later stages of “white flowers” and “rubellis flowers.” (a) Numbers of DEGs. (b) Venn diagram of DEGs. WE: “white flower” at early stage (stage 3); WL: “white flower” at later stage (stage 6); RE: “Rubellis flower” at early stage (stage 3); RL: “Rubellis flower” at later stage (stage 6).

Clean data from the above four libraries were normalized to FPKM values to calculate the expression of unigenes in *M. maudiae*. Unigenes that are differentially expressed in pairs of two stages of different color strains are shown in Figure 2. As the flower develops from the closed bud of stage 3 to stage 6, 19,581 and 28,002 unigenes are differentially expressed in white and rubellis flowers, respectively, indicating significant variation in gene expression profiles during the blooming of flower development. Specifically, the comparison of WE and RE and WL and RL resulted in 29,799 and 28,858 DEGs, respectively.

### 3.4. Analysis of DEGs Related to Phenylpropanoid, Flavonoid, and Anthocyanidin Biosynthetic Pathways in *M. maudiae*

**Figure 3:**
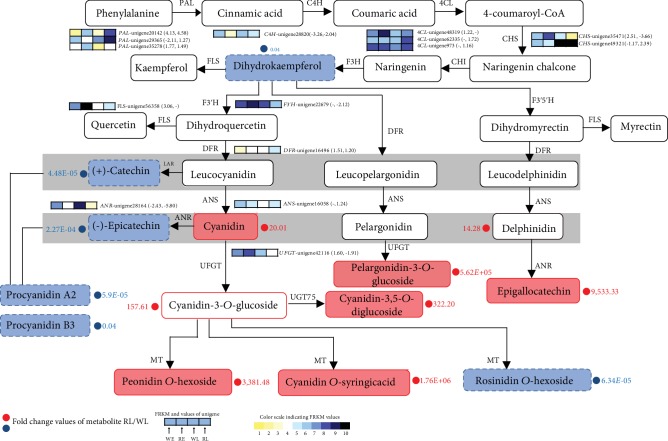
Metabolomic and transcript profiling in the phenylpropanoid and flavonoid biosynthetic pathways in “white flower” and “rubellis flower.” Grids with a color-scale from yellow to black represent FPKM values 1-10, 10-20, 20-40, 40-80, 80-160, 160-320, 320-640, 640-1,280, 1,280-2,560, and over 2,560, respectively. The numbers in square brackets after the grid represent log_2_-fold values of unigenes WE/RE and WL/RL, respectively. Compared with WL, in RL upregulated metabolites were in red boxes and downregulated metabolites were in blue boxes. PAL: phenylalanine ammonia-lyase; C4H: cinnamic acid 4-hydroxylase; 4CL: 4-coumarate-CoA ligase; CHS: chalcone synthase; CHI: chalcone isomerase; F3H: flavanone 3-hydroxylase; F3′H: flavanone 3′-hydroxylase; F3′5′H: flavanone 3′5′-hydroxylase; DFR: dihydroflavonol 4-reductase; ANS: anthocyanidin synthesis; UFGT: UDP-glucose flavonoid 3-*O*-glucosyltransferase; FLS: flavonol synthase; ANR: anthocyanidin reductase.

The increase in the contents of anthocyanins in rubellis flowers let us focus on the transcriptional changes in the phenylpropanoid, flavonoid, and anthocyanidin biosynthesis pathways that are related to flower pigmentation. Most of the biosynthesis pathways were strengthened by the upregulation of gene expression at the later stage of rubellis flowers (Figure 3). For the comparison of WE and RE, the transcript abundance of genes *PAL* (unigene20142, unigene35278), *4CL* (unigene48319), *CHS* (unigene35471), *FLS* (unigene56358), *DFR* (unigene 16496), and *UFGT* (unigene42116) were markedly upregulated in rubellis tepals than in white tepals, while one *CHS*-like gene (unigene49321), one *PAL* (unigene29365), and one anthocyanidin reductase (*ANR*, unigene28164) were downregulated in the rubellis tepals. At the later stage, the levels of transcripts encoding the secondary metabolite biosynthesis enzymes, including three *PAL* (unigene20142, unigene29365, and unigene35278), two *4CL* (unigene62335, unigene973), one *CHS* (unigene49321), one *DFR* (unigene16496), and one *ANS* (unigene16058), were markedly abundant in rubellis tepals compared with those in white tepals (Figure 3). It is noteworthy that the transcripts of *DFR*-like sequences presented higher levels of gene transcripts in rubellis tepals during the blooming of flowers (Figure 3). Notably, unigene28164 with an *ANR*-like sequence was downregulated 2.43-fold and 5.80-fold in the rubellis flower in early and later stages, respectively.

### 3.5. Transcription Factor Analyses

The TFs play an important role in regulating the expression of the structural genes during anthocyanin biosynthesis [33, 34]. Putative TFs were searched in the *M. maudiae* transcripts by HMMER software using the “Plant Transcription Factor Database” (http://planttfdb.cbi.pku.edu.cn/). Setting FDR < 0.0001 together with the absolute value of log_2_ ratio ≥ 2 as thresholds for significant differences, 120 and 105 genes were identified as TFs in WE vs. RE and WL vs. RL, respectively. And there were 105 and 161 differentially expressed TFs identified in WE vs. WL and RE vs. RL, respectively ([Supplementary-material supplementary-material-1]). The differentially expressed TFs here contained the family *MYB*, *bHLH*, *AP2*, *ERF*, *WRKY*, and *HSF*.

**Figure 4:**
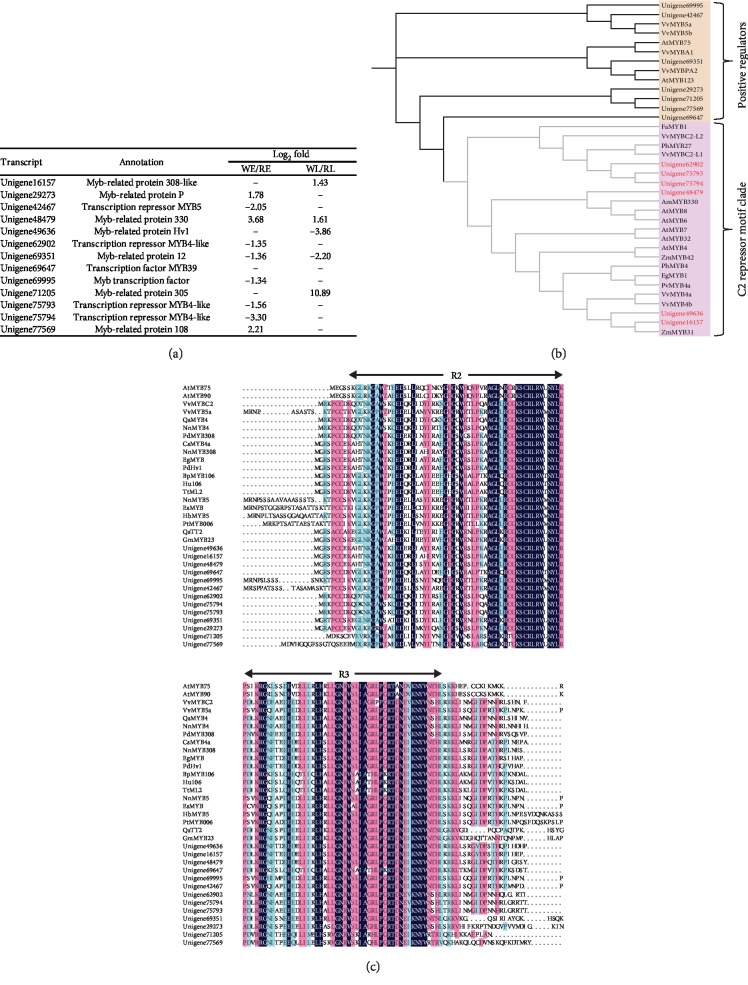
Changes in transcript abundance of predicted R2R3-MYB transcription factors. (a) Differentially expressed R2R3-MYBs in comparison groups. (b) Phylogenetic tree showing selected plant R2R3-MYB members. Accession numbers are listed in Materials and Methods. (c) Consensus sequences of R2R3 motif of MYB members.

Nine and five *R2R3-MYB*s were differentially expressed in WE vs. RE and WL vs. RL, respectively (Figure 4(a)). Among the *MYB*s in the comparison of WE and RE, three genes were identified upregulated and six downregulated in RE. In the later stage of flower development, two genes were found upregulated and three downregulated in RE. We found one *MYB* DEG (unigene48479) expressed at very high levels with FPKM ≥ 470 and FPKM ≥ 360 in RE and RL, respectively, but with low FPKM values in WE and WL. *R2R3-MYB*s also operate as positive and negative regulators. Six *R2R3-MYB*s were closely related to the C2 repressor motif clade in phylogenetic analyses (Figure 4(b)), which might play a key role in negatively regulating the level of anthocyanins. These *R2R3-MYB* proteins from the C2 repressor motif clade all have the EAR repression domain ([Supplementary-material supplementary-material-1]).

Twenty-five *bHLH* were found in WE vs. RE (13 upregulated, 12 downregulated, [Supplementary-material supplementary-material-1]). Twenty-six *bHLH* were markedly different in WL vs. RL (15 upregulated, 11 downregulated). For the comparison of WE vs. WL, 31 *bHLH* revealed differences, including 8 upregulated and 23 downregulated. During flower blooming, 51 *bHLH* DEGs were screened in RE vs. RL with 32 downregulated and 19 upregulated. Among these *bHLH* DEGs, 6 (unigene47744, unigene49005, unigene55012, unigene55013, unigene9769, and unigene54696) transcripts had markedly higher levels in both WE vs. RE and WL vs. RL. 4 (unigene95320, unigene6259, unigene41244, and unigene10054) *bHLH* DEGs had decreasing expression in both WE vs. RE and WL vs. RL.

### 3.6. RT-qPCR Validation of the Transcriptomic Data

**Figure 5:**
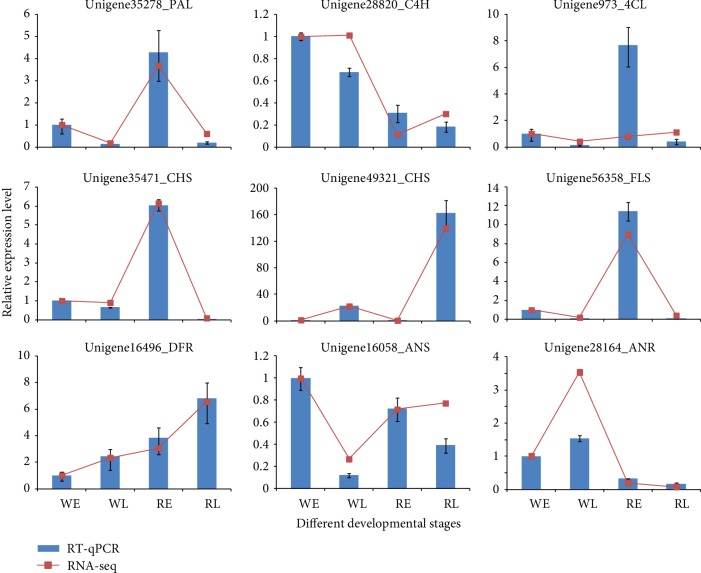
Real-time quantitative PCR validation of transcript profiles for representative color-related genes. Values for mean expression and standard deviation were calculated from the results of three independent replicates.

To validate the transcriptome results, nine color-related DEGs were selected for RT-qPCR analysis. The expression patterns of these genes were very similar to the sequencing results (Figure 5). A significant correlation (*R* > 0.84) was found between the two methods, indicating that the relevance of the RNA-seq data and RT-qPCR showed good consistency ([Supplementary-material supplementary-material-1]).

## 4. Discussion

Color mutations play an important role in plant breeding and horticulture. The complex metabolic mechanism of the mutants is beginning to be revealed using metabolomic and high-throughput sequencing methods [11]. In this study, the combined analysis of metabolites and transcriptomes of two *M. maudiae* that differ in color was carried out, providing large-scale information on metabolic product profiles and the underlying modifications in gene expression profiles.

Flower development and pigmentation are tightly linked with anthocyanin biosynthesis in most cases [35, 36]. The primary shade of flower color is mainly determined by three biosynthesis reactions: pelargonidin, delphinidin, and cyanidin [6]. Herein, most derivatives of anthocyanidins were highly significantly accumulated in the rubellis flowers, while the colorless procyanidin and other catechin derivatives were highly accumulated in white flowers. This finding provides a global view of the large-scale secondary metabolite changes for color maturation in *M. maudiae*. Although the level of anthocyanins was limited, tepal extracts of white flowers contained all of the most core anthocyanidin compositions that had been detected in rubellis flowers, including the derivatives of pelargonidin, delphinidin, and cyanidin. This suggested that the lack of anthocyanin in white tepals cannot be due to any blockage of the corresponding reactions. There might be multiple reasons for the loss of pigmentation. The cyanidin anthocyanins mainly tend to exist as a form of 3,5-*O*-diglucoside, *O*-syringic acid, and 3-*O*-glucoside and peonidin *O*-hexoside in rubellis tepals, while they were mainly present in the white-flowered strain with rosinidin *O*-hexoside, the colorless procyanidin, and other catechin derivatives. Cyanidin derivatives have been identified as being the major anthocyanins present in the skin of red apples, and also in the purplish-red, bronze, and pink *Chrysanthemum* inflorescences [37, 38]. Hence, we inferred that cyanidin and cyanidin derivatives might be the target compound for determining the pigmentation of the rubellis tepals in *M. maudiae*. Surprisingly, the levels of one derivative of cyanidin, rosinidin *O*-hexoside, were markedly higher in white tepals but were barely detectable in the rubellis flower strain. Rosinidin has been found in *Primula rosea* [39] and *Catharanthus roseus* [40] as a pigment. Further studies are needed to determine the role of rosinidin *O*-hexoside in *M. maudiae*. Catechin and epicatechin were increasingly expressed in white tepals. The units of catechin and epicatechin can compose proanthocyanidins, an important quality component of many fruits [41]. The levels of all of the proanthocyanidins that were identified in rubellis tepal extracts were markedly higher in white tepals. It could be concluded that the middle-stream metabolites in the leucoanthocyanidins or biosynthetic anthocyanidins were also present in the white tepals but were mainly converted to colorless proanthocyanidins.

In the synthesis of pelargonidin, the color compound peloargonidin-3-*D*-glucoside was abundant in rubellis tepals. In the synthesis of delphinidin, the increasing expression of epigallocatechin in rubellis tepals might partially block the synthesis of color delphinidin derivatives and cause the production of other colorless compounds. It has been explained why blue is not the predominant color hue in *M. maudiae*. It is the conclusion that the metabolism of cyanidin plays a vital role in the flower coloration system of *M. maudiae*, whereas the metabolism of pelargonidin and delphinidin is less significant, resulting in the appearance of rubellis pigments.

For other metabolite compounds, several forms of tricin were markedly upregulated in rubellis flowers. Tricin is widely recognized as a valuable health compound due to its anti-inflammatory, antioxidant, and cardioprotective potentials [42]. The biosynthesis of tricin starts from naringenin and produces apigenin, which requires further modifications to generate tricin. Apigenin is converted to luteolin by *F3*′*H* and then to chrysoeriol by an OMT. Chrysoeriol generates selgin and then sequentially leads to tricin formation [43]. The lower expression of *F3*′*H* was found in white tepals and might result in the low content of the derivatives of tricin. Recent studies mainly focused on the role of tricin in lignification [43, 44]. Further studies are needed to determine the role of tricin *O*-linked conjugates in pigmentation.

Genes *PAL*, *C4H*, *4CL*, *CHS*, *F3H*, *DFR*, *F3*′*H*, *ANR*, *FLS*, and *ANS* in phenylpropanoid and flavonoid biosynthesis pathways were identified in the transcriptome. Most of these genes were upregulated in rubellis tepal, except *ANR*, *F3*′*H*, and *UFGT*. It is notable that the level of gene transcripts of *DFR*-like sequences was higher in rubellis flowers not only in the early stage but also in the later stage. *DFR* reduces dihydroflavonols to colorless leucoanthocyanidins, which are catalyzed by *ANS* to colored anthocyanidin. The suppression of the *DFR* expression in *Dianthus caryophyllus* L. or the low expression in *Arabidopsis* restricted their pigmentation [45, 46]. The transcripts of *DFR* were abundant in rubellis tepals, but only a trace of expression was detected in the white tepals, suggesting that *DFR* may be important for pigmentation probably through a quantitative regulatory system rather than through the absolute blockage of anthocyanin biosynthesis, similar to the coloration of *Chrysanthemum* flowers [2]. Recently, studies have shown that the overexpression of *McDFR* increased anthocyanin production, resulting in red-leaf and red fruit peel phenotypes in crabapples (*Malus* spp.) [47]. The upregulation of *DFR* could increase the production of anthocyanins in rubellis tepals. Another enzyme, ANS, was encoded by unigene16058_All, which shared the highest identities (99%) with the *ANS* gene of *Magnolia sprengeri* in GenBank. ANS, one of the dioxygenases, can catalyze the formation of colored anthocyanidins from leucoanthocyanidins, which might result in the accumulation of anthocyanin. So if *ANS* reactions are strongly enhanced, the reaction of colored anthocyanin production is effectively positive. One *ANR* (unigene28164) was increasingly expressed in white flowers. The introduction of *ANR* genes into tobacco (*Nicotiana tabacum* cv. Petite Havana SR1) resulted in the loss of anthocyanins and in the accumulation of catechin and epicatechin contents [48]. Transcript accumulation of *ANR* genes and the abundance of the content of catechin and epicatechin in white flowers in our result correspond well with this phenomenon in tobacco transgenic flowers. Proanthocyanidins are phenolic polymers of condensed flavan-3-ols and are the major compounds in higher plants. Overexpression of *ANR* genes in tobacco have been reported to promote the biosynthesis of proanthocyanidins in flowers [48]. What's more, a negative correlation between anthocyanin and proanthocyanidin biosynthesis was also found during leaf and fruit peel development in *Malus* crabapple [49]. Similarly, the levels of two types of proanthocyanidins (Procyanidins A2 and B3) were significantly higher in white flowers than those of the rubellis flowers.

Plants require complex regulatory mechanisms to make sure that the level of anthocyanin pigmentation is appropriate for environmental changes. The degree of anthocyanins is transcriptionally controlled by positive and negative regulators from the MYB, bHLH, and WD repeat (MBW) families. Three *R2R3-MYB* members (unigene62902, unigene75793, and unigene75794) that clustered with VvMYBC2-L1 and PhMYB27 were expressed at lower levels in RE. In *V. vinifera*, VvMYB4a and VvMYB4b showed a severe reduction in phenolic compounds, and VvMYBC2-L1 and VvMYBC2-L3 may play a key role in negatively regulating the flavonoid biosynthesis, balancing the inductive effects of positive regulators [50]. Experiments with *P. hybrida* indicate that PhMYB27 can act as an anthocyanin repressor and repress transcription through its EAR repression domain [51]. Thus, unigene62902, unigene75793, and unigene75794 might be closely related to color morph regulation in *M. maudiae*. Unigene48479 was closely related to AmMYB330, AtMYB6, and AtMYB8. Overexpression of *AmMYB330* from the flowers of *Antirrhinum* has been proven to inhibit phenylpropanoid metabolism in transgenic tobacco (*Nicotiana tabacum* cv Samsun NN) plants [52]. Unigene49636 and unigene16157 clustered with ZmMYB31, which showed a significantly reduced lignin content and negatively regulated several genes in the phenylpropanoid pathway [53]. This illustrates that unigene48479, unigene49636, and unigene16157 might be related to the regulation of the phenylpropanoid biosynthesis in *M. maudiae*.

## 5. Conclusions

Our metabolomic and transcriptomic data provide novel insights in understanding the modulated metabolites and gene expression in the rubellis mutant. Differentially altered metabolites associated with flower pigmentation were identified between white and rubellis tepals. Putative homologues of color-related genes involved in the phenylpropanoid and flavonoid biosynthesis pathways were identified in the transcriptome. The changes of anthocyanins and the discovery of genes associated with their biosynthesis and metabolism in *M. maudiae* are interesting and necessary for further studies.

## Figures and Tables

**Table 1:** Differentially altered metabolite compounds in the tepals of the later stage of *M. maudiae*.

Class	Compounds	Average content		VIP	Fold change
		WL	RL		
Anthocyanins	Peonidin *O*-hexoside	9	3.04*E* + 04	1.65	3381.48
	Rosinidin *O*-hexoside	1.42*E* + 05	9	1.81	6.34*E* − 05
	Cyanidin 3-*O*-glucoside	3.37*E* + 05	5.32*E* + 07	1.31	157.61
	Cyanidin *O*-syringic acid	9	1.58*E* + 07	2.21	1.76*E* + 06
	Cyanidin 3,5-*O*-diglucoside	3.99*E* + 03	1.29*E* + 06	1.40	322.20
	Pelargonidin 3-*O*-glucoside	9	5.06*E* + 06	2.12	5.62*E* + 05
	Cyanidin	6.58*E* + 04	1.32*E* + 06	1.01	20.01

Catechin derivatives	Catechin-catechin-catechin	2.01*E* + 05	9	1.84	4.48*E* − 05
	Epicatechin-epiafzelechin	3.96*E* + 04	9	1.68	2.27*E* − 04
	Epigallocatechin	9	8.58*E* + 04	1.76	9533.33
	Protocatechuic aldehyde	9	1.82*E* + 05	1.83	2.03*E* + 04
	Protocatechuic acid	9	2.99*E* + 04	1.66	3318.52

Proanthocyanidins	Procyanidin A2	1.53*E* + 05	9	1.82	5.90*E* − 05
	Procyanidin B3	1.03*E* + 07	3.88*E* + 05	1.05	0.04

Flavonol	Kumatakenin	2.65*E* + 04	9	1.61	3.40*E* − 04
	Dihydrokaempferol	8.96*E* + 06	3.85*E* + 05	1.03	0.04
	Rhamnetin	7.23*E* + 03	1.66*E* + 06	1.35	229.24
	Fustin	1.15*E* + 07	5.61*E* + 05	1.01	0.05

Isoflavone	Calycosin	2.72*E* + 03	1.88*E* + 05	1.20	69.20
	Prunetin	1.61*E* + 04	3.39*E* + 05	1.01	21.06
	Sissotrin	1.05*E* + 04	9.42*E* + 05	1.23	89.94

Flavone	Selgin 5-*O*-hexoside	6.45*E* + 04	2.19*E* + 06	1.09	34.02
	*O*-Methylchrysoeriol 5-*O*-hexoside	8.55*E* + 03	1.96*E* + 06	1.36	229.63
	Tricin *O*-vanilloyl hexoside	9	2.70*E* + 04	1.65	3000.00
	*O*-Methylchrysoeriol 7-*O*-hexoside	1.03*E* + 04	2.09*E* + 06	1.34	202.46
	Tricetin *O*-rutinoside	9	1.80*E* + 04	1.60	2000.00
	Luteolin *O*-hexosyl-*O*-pentoside	8.44*E* + 05	3.68*E* + 04	1.03	0.04
	Chrysoeriol *O*-hexosyl-*O*-hexoside	9	6.60*E* + 04	1.74	7337.04
	Tricin 5-*O*-hexosyl-*O*-hexoside	3.94*E* + 03	6.41*E* + 05	1.61	162.72
	Tricin di-*O*-hexoside	9	3.17*E* + 06	2.08	3.52*E* + 05
	Tricin *O*-rhamnoside	1.68*E* + 03	1.85*E* + 06	1.78	1105.81
	Tricin *O*-hexosyl-*O*-syringin alcohol	9	3.30*E* + 04	1.67	3670.37
	Chrysoeriol 7-*O*-hexoside	1.90*E* + 06	9	2.04	4.73*E* − 06
	Luteolin *O*-eudesmic acid-*O*-hexoside	1.85*E* + 03	7.03*E* + 04	1.40	38.03
	Tricin 5-*O*-hexoside	9	4.21*E* + 06	2.10	4.68*E* + 05
	Chrysoeriol	5.00*E* + 05	1.33*E* + 04	1.11	0.03
	Acacetin	1.55*E* + 04	3.25*E* + 05	1.01	20.99
	Sakuranetin	4.68*E* + 03	2.07*E* + 05	1.12	44.26

Flavone *C*-glycosides	*C*-Pentosyl-*C*-hexosyl-apigenin	1.03*E* + 05	5.27*E* + 03	1.01	0.05
	*C*-Hexosyl-apigenin *O*-caffeoylhexoside	2.89*E* + 04	9	1.65	3.12*E* − 04
	Luteolin *O*-feruloylhexoside	9	1.66*E* + 04	1.59	1840.74
	8-*C*-Hexosyl-apigenin *O*-hexosyl-*O*-hexoside	9	9.75*E* + 04	1.77	1.08*E* + 04
	*C*-Hexosyl-apigenin *O*-pentoside	4.06*E* + 04	2.38*E* + 03	1.05	0.06
	8-*C*-Hexosyl-luteolin *O*-hexoside	9	2.91*E* + 04	1.65	3229.63
	Chrysoeriol *C*-hexoside	9	1.90*E* + 04	1.61	2107.41
	Isovitexin	1.78*E* + 05	6.36*E* + 03	1.06	0.04

Flavanone	Naringenin *O*-malonylhexoside	2.22*E* + 03	1.77*E* + 04	1.19	7.96
	7-*O*-Methyleriodictyol	1.61*E* + 03	8.68*E* + 05	1.54	538.13

*Note*. WL: “white flower” at later stage; RL: “rubellis flower” at later stage. Number 9 represents a level so low as to barely be detectable. Differentially accumulated compounds were identified by threshold VIP (variable importance in projection) ≥ 1.

## Data Availability

All sequence data were deposited at the NCBI in the Sequence Read Archive (SRA) database under the accession numbers SRR8173854, SRR8173855, SRR8173856, and SRR8173857 for WE, WL, RE, and RL, respectively.

## Supplementary Materials

Supplementary 1 Figure S1: total content of anthocyanins detected by a spectrophotometer.

Supplementary 2 Figure S2: the metabolite profiles of a white flower and a rubellis flower at the later stage.

Supplementary 3 Figure S3: length distribution of sequencing reads and contigs of *M. maudiae*.

Supplementary 4 Figure S4: the top-hit species distribution of *M. maudiae*.

Supplementary 5 Figure S5: histogram representing Gene Ontology (GO) classification of *M. maudiae*.

Supplementary 6 Figure S6: histogram representing Clusters of Orthologous Groups of proteins (COG) classification of all unigenes in *M. maudiae*.

Supplementary 7 Figure S7: consensus sequences of C2 repressor motif clade identified by MEME Suite.

Supplementary 8 Figure S8: correlation of the expression levels of nine differentially expressed genes by RNA-seq and RT-qPCR analysis.

Supplementary 9 Table S1: developmental stages of flowers in *M. maudiae*.

Supplementary 10 Table S2: primers used in quantitative real-time PCR.

Supplementary 11 Table S3: summary of RNA-seq results.

Supplementary 12 Table S4: number of differentially expressed transcription factors in comparison groups.
