# Supplementary figures and images for: Integrated Metabolome and Transcriptome Analysis Uncovers the Role of Anthocyanin Metabolism in Michelia maudiae
Source: Int J Genomics. 2019 Nov 3;2019:4393905. doi: 10.1155/2019/4393905 (PMC6874964; doi:10.1155/2019/4393905)

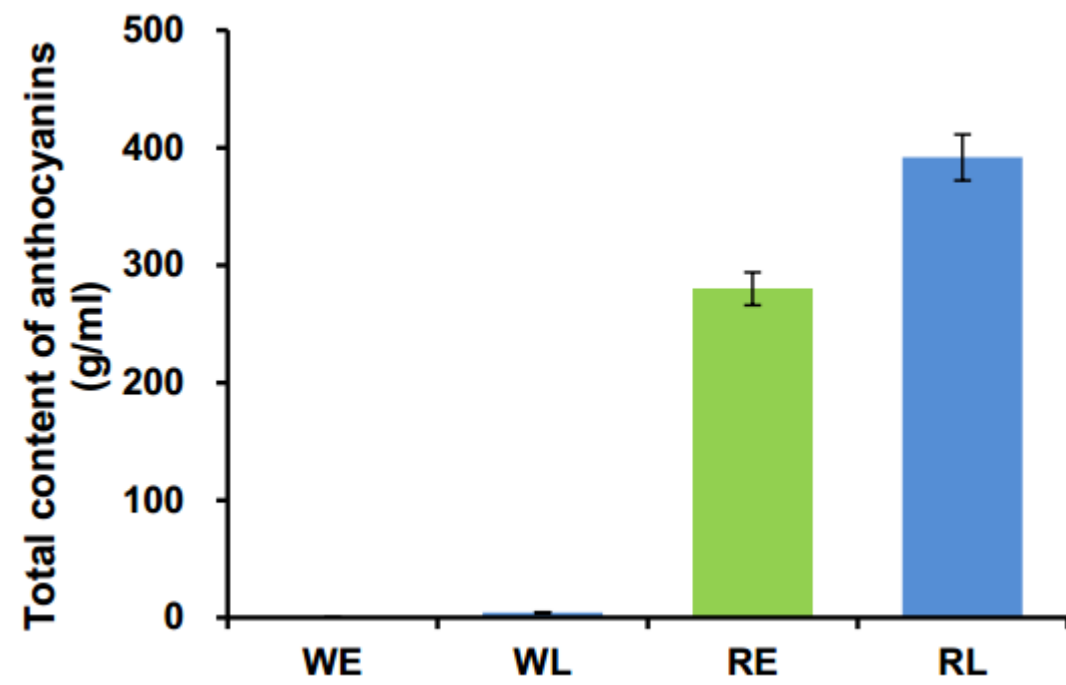

Supplement: Supplementary 1 — Figure S1: total content of anthocyanins detected by a spectrophotometer. [file 4393905.f1.pdf]

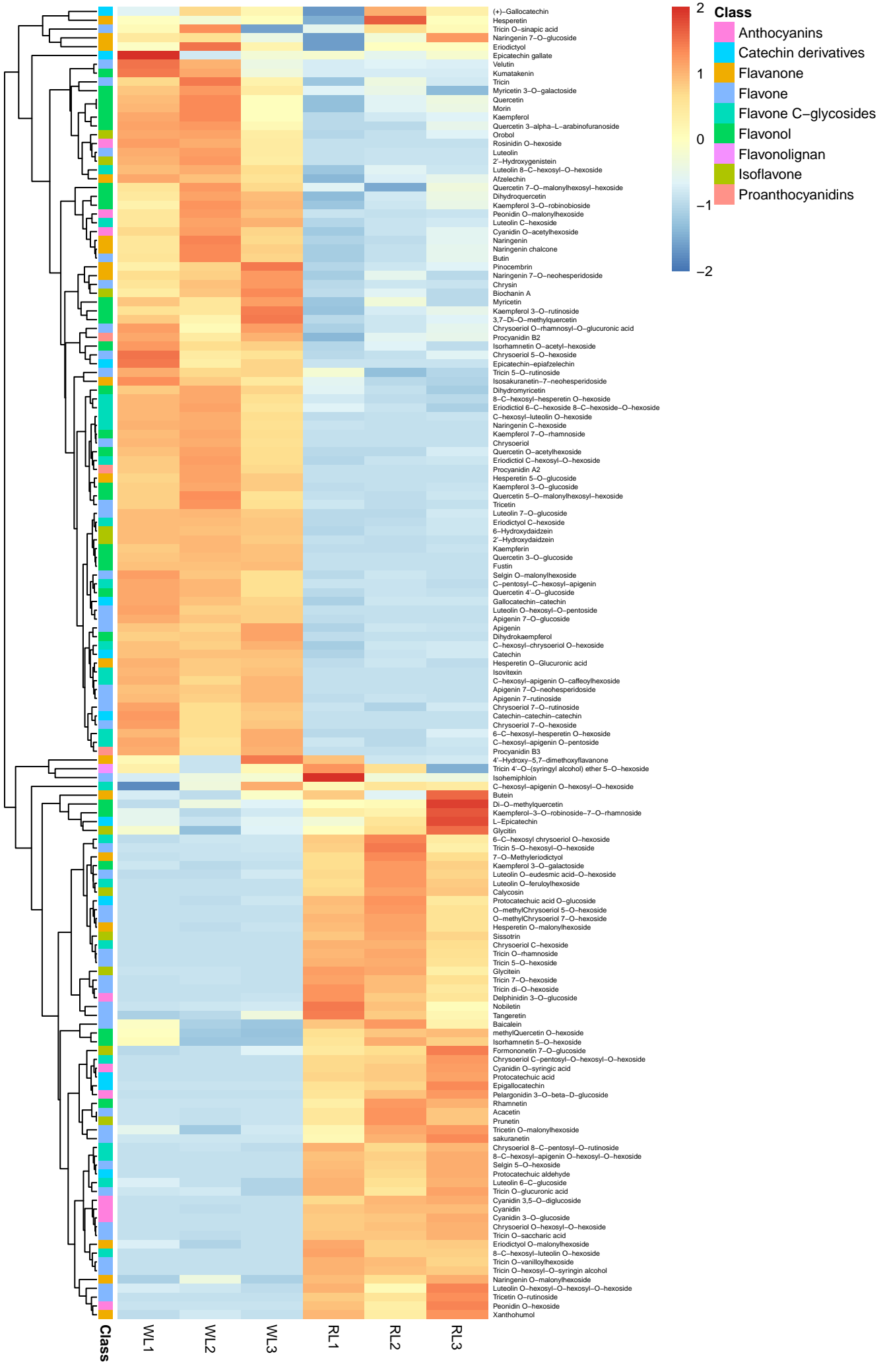

Supplement: Supplementary 2 — Figure S2: the metabolite profiles of a white flower and a rubellis flower at the later stage. [file 4393905.f2.pdf]

# All-Unigene Length Distribution

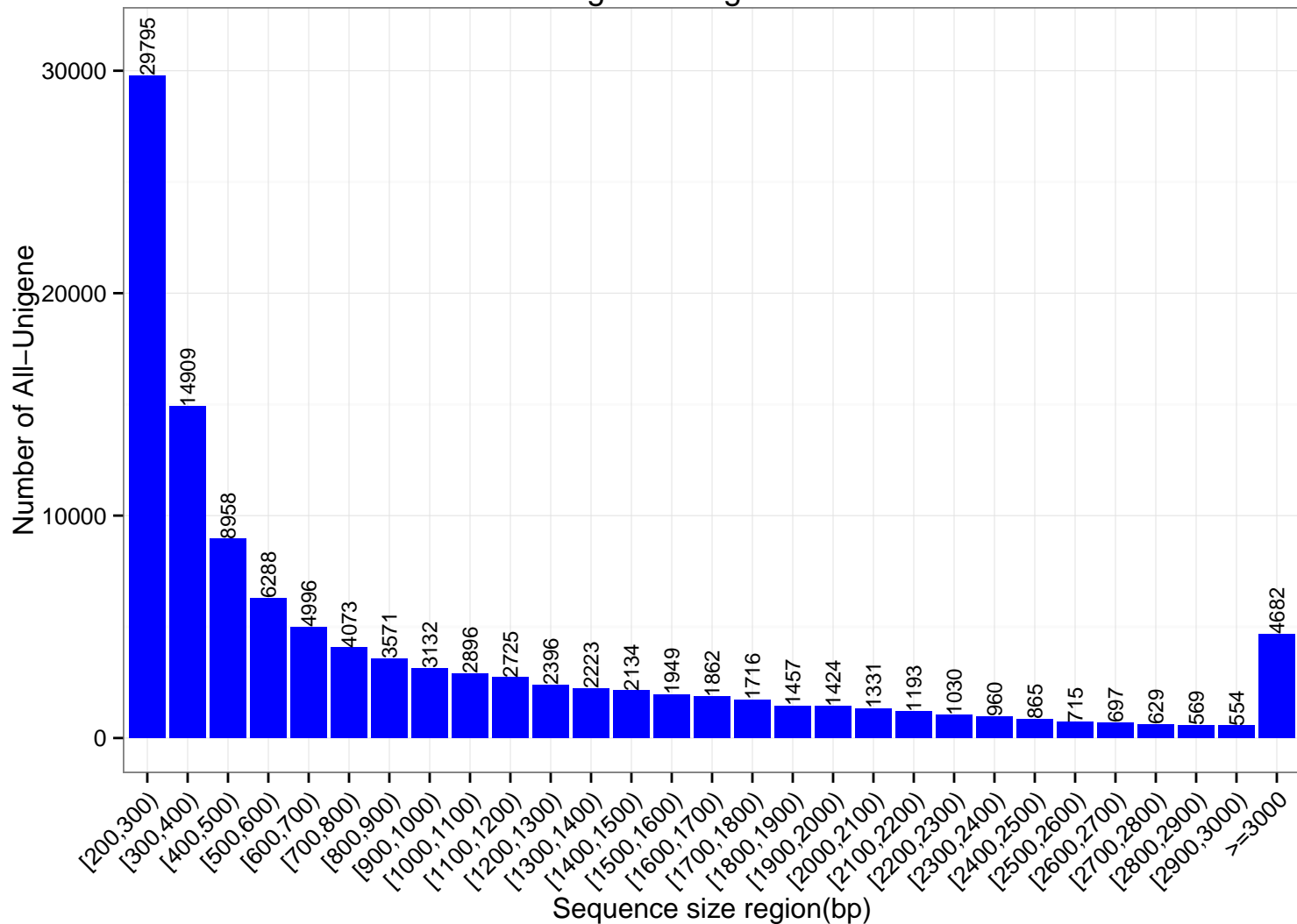

Supplement: Supplementary 3 — Figure S3: length distribution of sequencing reads and contigs of M. maudiae. [file 4393905.f3.pdf]

# The top hit species distribution

Species

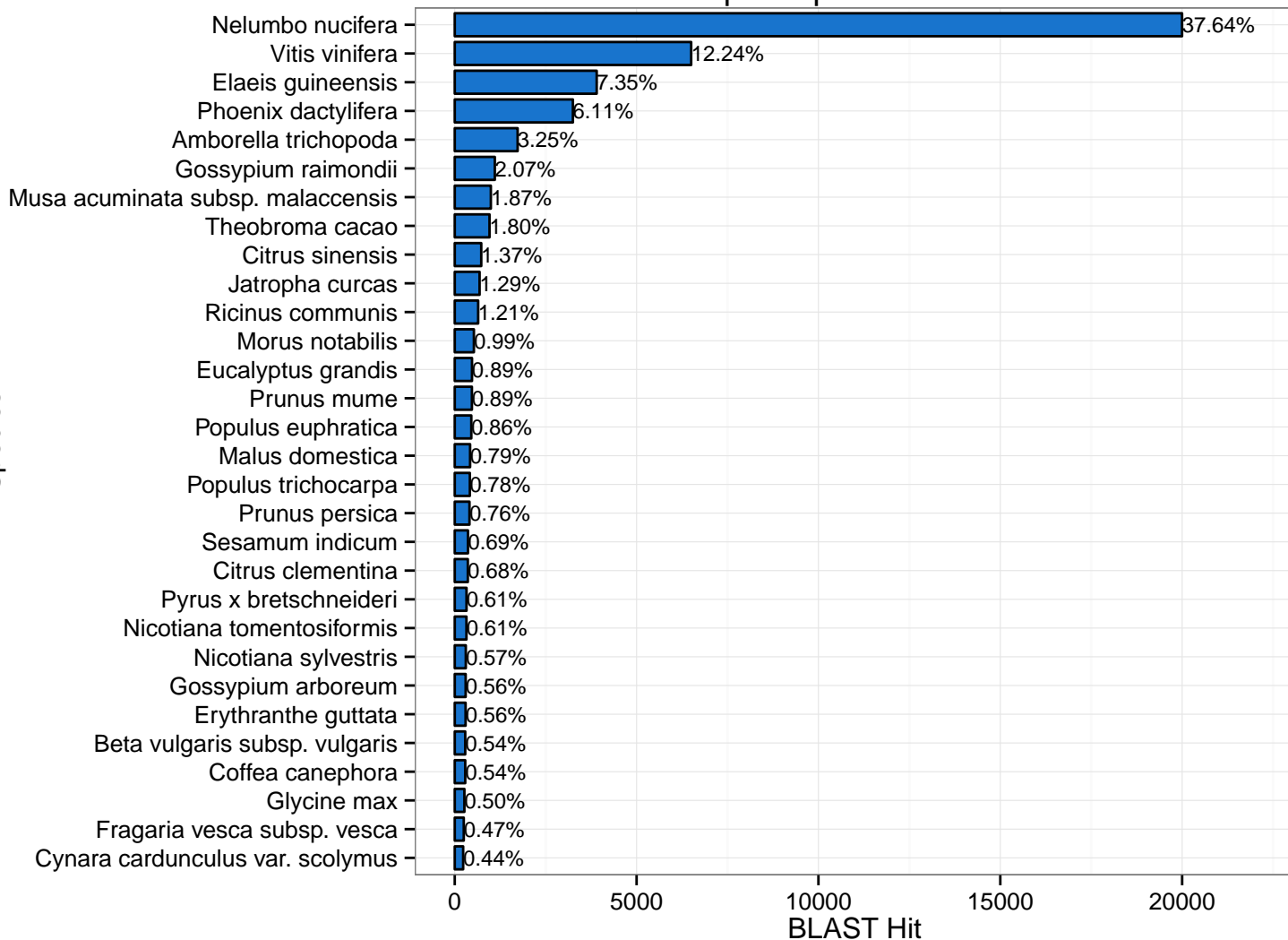

Supplement: Supplementary 4 — Figure S4: the top-hit species distribution of M. maudiae. [file 4393905.f4.pdf]

# All-Unigene GO Classification

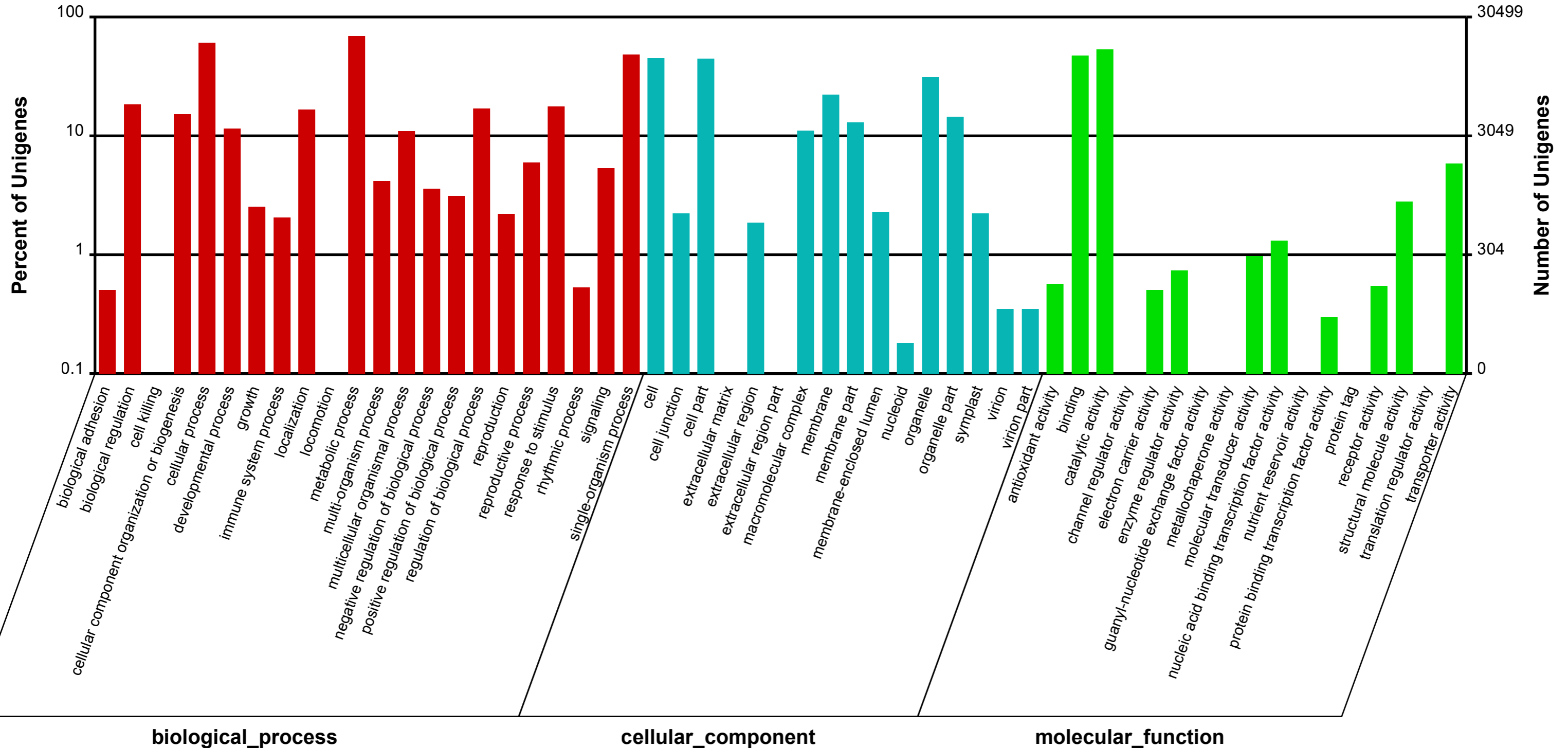

Supplement: Supplementary 5 — Figure S5: histogram representing Gene Ontology (GO) classification of M. maudiae. [file 4393905.f5.pdf]

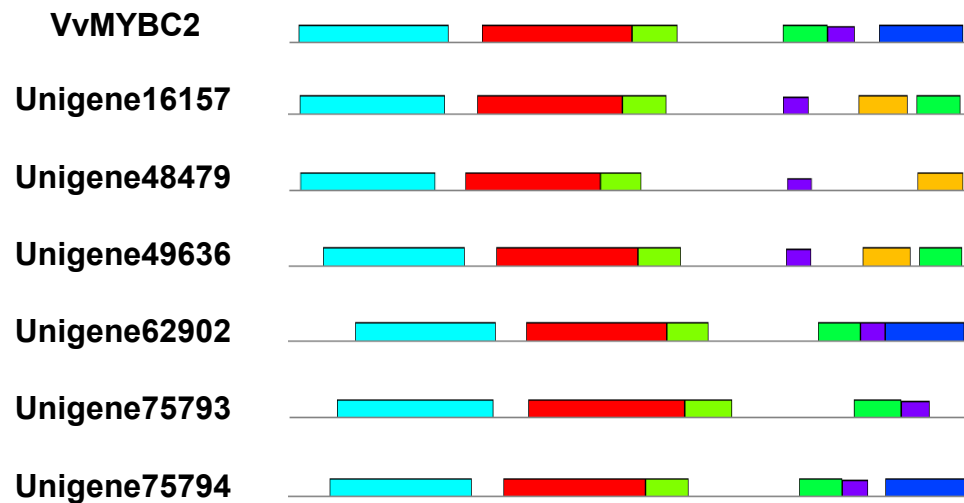

**R2 motif**

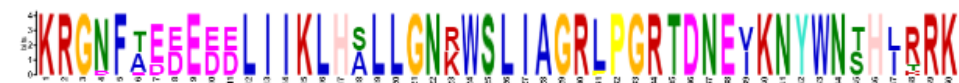

**R3 motif**

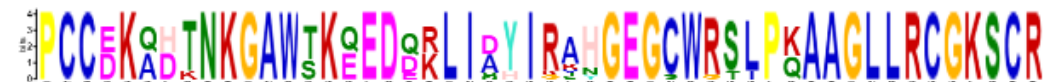

**C1**

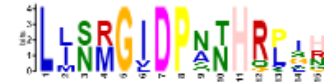

**C2 / EAR-repressor motif**

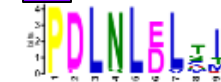

**C3 / Zinc finger-like motif**

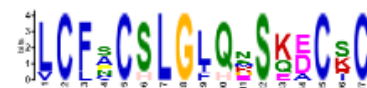

**C4**

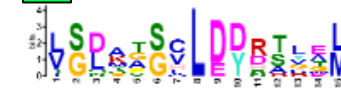

**C5 / TLLLFR-repressor motif**

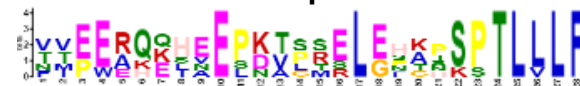

Supplement: Supplementary 7 — Figure S7: consensus sequences of C2 repressor motif clade identified by MEME Suite. [file 4393905.f7.pdf]

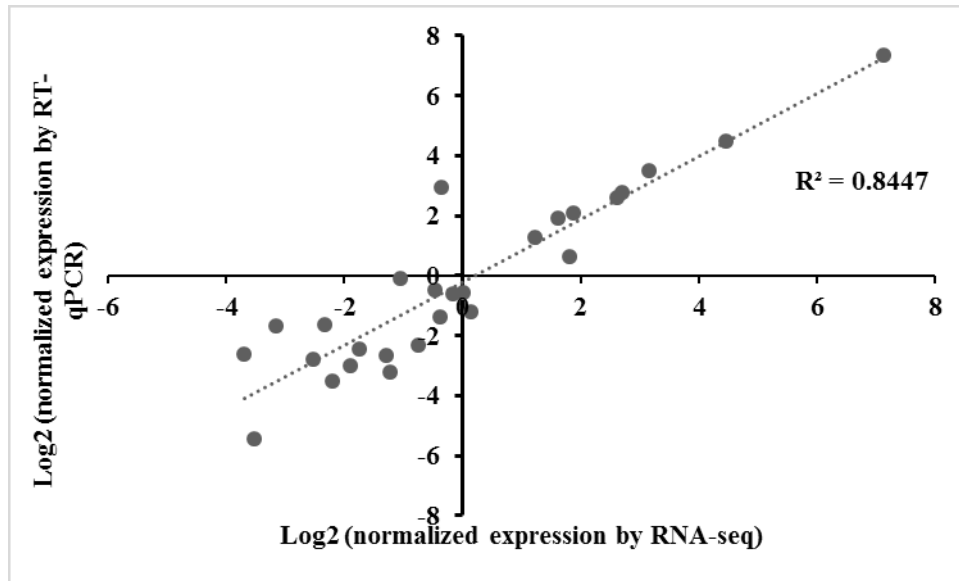

Supplement: Supplementary 8 — Figure S8: correlation of the expression levels of nine differentially expressed genes by RNA-seq and RT-qPCR analysis. [file 4393905.f8.pdf]
