# Supplementary material for: Integrated Metabolome and Transcriptome Analysis Uncovers the Role of Anthocyanin Metabolism in Michelia maudiae
Source: Int J Genomics. 2019 Nov 3;2019:4393905. doi: 10.1155/2019/4393905 (PMC6874964; doi:10.1155/2019/4393905)
Supplement: Supplementary 6 — Figure S6: histogram representing Clusters of Orthologous Groups of proteins (COG) classification of all unigenes in M. maudiae. [file 4393905.f6.pdf]

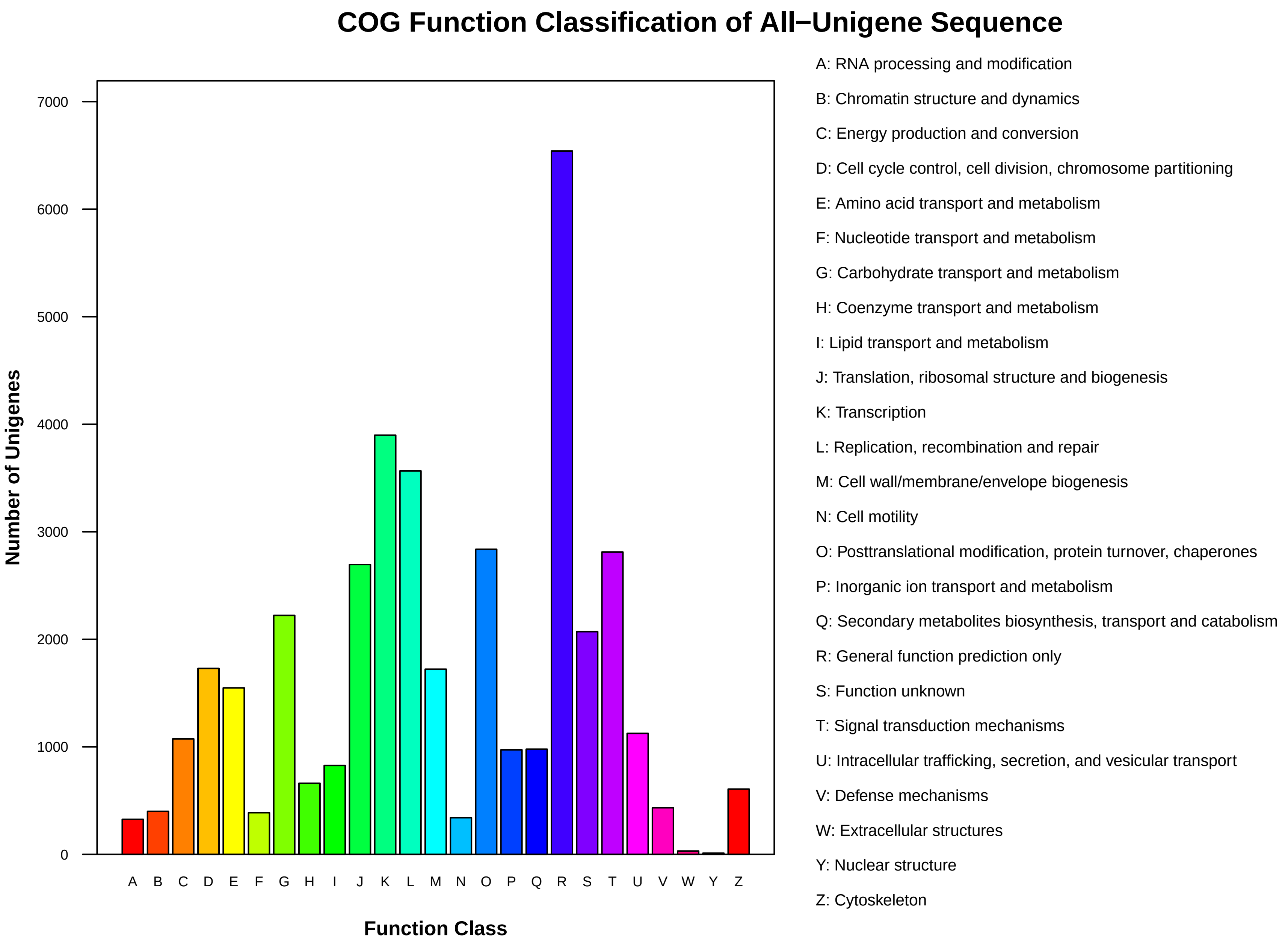
COG Function Classification of All−Unigene Sequence
Number of Unigenes
0
1000
2000
3000
4000
5000
6000
7000
A B C D E F G H I J K L M N O P Q R S T U V W Y Z
Function Class
A: RNA processing and modification
B: Chromatin structure and dynamics
C: Energy production and conversion
D: Cell cycle control, cell division, chromosome partitioning
E: Amino acid transport and metabolism
F: Nucleotide transport and metabolism
G: Carbohydrate transport and metabolism
H: Coenzyme transport and metabolism
I: Lipid transport and metabolism
J: Translation, ribosomal structure and biogenesis
K: Transcription
L: Replication, recombination and repair
M: Cell wall/membrane/envelope biogenesis
N: Cell motility
O: Posttranslational modification, protein turnover, chaperones
P: Inorganic ion transport and metabolism
Q: Secondary metabolites biosynthesis, transport and catabolism
R: General function prediction only
S: Function unknown
T: Signal transduction mechanisms
U: Intracellular trafficking, secretion, and vesicular transport
V: Defense mechanisms
W: Extracellular structures
Y: Nuclear structure
Z: Cytoskeleton
